# Supplementary material for: Substantial differences in source contributions to carbon emissions and health damage necessitate balanced synergistic control plans in China
Source: Nat Commun. 2024 Jul 13;15:5880. doi: 10.1038/s41467-024-50327-8 (PMC11245606; doi:10.1038/s41467-024-50327-8)
Supplement: Supplementary file 1 — Supplementary Information [file 41467_2024_50327_MOESM1_ESM.pdf]

Supplementary Information for

Substantial differences in source contributions to carbon emissions and health damage necessitate balanced synergistic control plans in China

Yilin Chen[1,2*], Huizhong Shen[2,3], Guofeng Shen[4], Jianmin Ma[4], Yafang Cheng[5], Armistead G. Russell[6], Shunliu Zhao[7], Amir Hakami[7], Shu Tao[2,4]

[1]School of Urban Planning and Design, Peking University Shenzhen Graduate School, Shenzhen 518055, China

[2]Shenzhen Key Laboratory of Precision Measurement and Early Warning Technology for Urban Environmental Health Risks, School of Environmental Science and Engineering, Southern University of Science and Technology, Shenzhen 518055, China

[3]Guangdong Provincial Observation and Research Station for Coastal Atmosphere and Climate of the Greater Bay Area, School of Environmental Science and Engineering, Southern University of Science and Technology, Shenzhen 518055, China

[4]College of Urban and Environmental Sciences, Peking University, Beijing 100871, China

[5]Max Planck Institute for Chemistry, Mainz 55128, Germany

[6]School of Civil and Environmental Engineering, Georgia Institute of Technology, Atlanta Georgia 30332, United States

[7]Department of Civil and Environmental Engineering, Carleton University, Ottawa, Ontario K1S5B6, Canada

[*]Corresponding author, e-mail: ylchen2023@pku.edu.cn

This supplementary information includes supplementary tables 1-3, supplementary figures 1-10, and supplementary note 1.

Supplementary Table 1 The monetized health damage, CO_2 emissions, and integrated costs in US dollars, as well as their associated ranks for all source sectors assessed in this study

sector	fuel/process category	monetized health damages (rank)	monitized climate impacts (rank)	integrated costs (rank)
industry	bituminous coal	2.2×10^{11} (1)	2.6×10^{11} (2)	4.8×10^{11} (2)
	anthracite	9.8×10^{9} (22)	8.7×10^{9} (13)	1.9×10^{10} (20)
	coking coal	4.9×10^{9} (26)	5.3×10^{9} (16)	1.0×10^{10} (26)
	gas/diesel	2.6×10^{10} (16)	8.9×10^{9} (12)	3.5×10^{10} (15)
	dry natural gas	1.4×10^{9} (37)	9.0×10^{9} (11)	1.0×10^{10} (24)
	crude oil	3.4×10^{9} (31)	9.2×10^{9} (10)	1.3×10^{10} (22)
	residue fuel oil	1.1×10^{9} (39)	2.6×10^{9} (21)	3.6×10^{9} (35)
	industrial waste	1.5×10^{4} (53)	6.2×10^{4} (36)	7.7×10^{4} (53)
	primary Al production	1.9×10^{9} (33)	2.0×10^{9} (22)	3.9×10^{9} (34)
	hydraulic cement production	1.8×10^{11} (5)	7.6×10^{10} (4)	2.5×10^{11} (4)
	iron and steel production	1.3×10^{11} (7)	1.2×10^{11} (3)	2.4×10^{11} (5)
	coke production	5.7×10^{10} (10)	3.6×10^{7} (7)	9.4×10^{10} (8)
	brick production	2.7×10^{10} (15)	1.2×10^{10} (9)	3.9×10^{10} (13)
	petroleum catalytic cracking	1.6×10^{9} (36)	0 (N/A)	1.6×10^{9} (38)
	lime production	2.8×10^{10} (14)	0 (N/A)	2.8×10^{10} (18)
	glass production	5.3×10^{9} (25)	0 (N/A)	5.3×10^{9} (32)
	fertilizer production	1.3×10^{10} (20)	0 (N/A)	1.3×10^{10} (21)
	ferroalloy production	8.9×10^{9} (23)	0 (N/A)	8.9×10^{9} (29)
	lead production	8.7×10^{8} (42)	0 (N/A)	8.7×10^{8} (45)
	magnesium production	1.6×10^{8} (48)	0 (N/A)	1.6×10^{8} (49)
	zinc production	1.1×10^{9} (38)	0 (N/A)	1.1×10^{9} (42)
	ammonia production	1.6×10^{9} (35)	0 (N/A)	1.6×10^{9} (37)
	nickel and copper production	4.2×10^{9} (28)	0 (N/A)	4.2×10^{9} (33)
	natural gas production	1.2×10^{10} (21)	0 (N/A)	1.2×10^{10} (23)
energy generation	bituminous coal	1.8×10^{11} (4)	3.5×10^{11} (1)	5.3×10^{11} (1)
	coking coal	2.6×10^{7} (51)	5.1×10^{7} (33)	7.6×10^{7} (52)
	gas/diesel	6.6×10^{7} (50)	1.7×10^{8} (31)	2.4×10^{8} (48)
	dry natural gas	2.3×10^{9} (32)	8.0×10^{9} (14)	1.0×10^{10} (25)
	residue fuel oil	3.6×10^{8} (46)	1.1×10^{9} (26)	1.5×10^{9} (39)
	solid biomass	2.1×10^{8} (47)	6.5×10^{8} (27)	8.6×10^{8} (46)

Supplementary Table 1 The monetized health damage, CO_2 emissions, and integrated costs in US dollars, as well as their associated ranks for all source sectors assessed in this study (continued)

sector	fuel/process category	monetized health damages (rank)	monitized climate impacts (rank)	integrated costs (rank)
domestic	bituminous coal	2.1×10^{11} (2)	2.6×10^{10} (8)	2.3×10^{11} (6)
	honeycomb/coal briquettes	2.9×10^{10} (13)	3.7×10^{9} (18)	3.2×10^{10} (16)
	indoor firewood burning	5.0×10^{10} (11)	3.0×10^{9} (20)	5.3×10^{10} (12)
	non-organized waste burning	9.3×10^{8} (41)	2.4×10^{7} (34)	9.5×10^{8} (43)
	patent fuel	8.8×10^{9} (24)	1.2×10^{9} (25)	1.0×10^{10} (27)
	gas/diesel	1.8×10^{9} (34)	1.4×10^{9} (23)	3.2×10^{9} (36)
	dry natural gas	1.0×10^{9} (40)	2.3×10^{8} (30)	1.2×10^{9} (41)
	liquid petroleum gas	3.7×10^{9} (30)	6.1×10^{9} (15)	9.8×10^{9} (28)
	gas works gas	8.6×10^{8} (43)	4.9×10^{8} (28)	1.4×10^{9} (40)
	coke oven gas	6.1×10^{8} (44)	3.4×10^{8} (29)	9.5×10^{8} (44)
	biogas	1.4×10^{7} (52)	8.3×10^{7} (32)	9.6×10^{7} (51)
	indoor crop residue burning	6.4×10^{10} (8)	0 (N/A)	6.4×10^{10} (9)
	charcoal	4.2×10^{8} (45)	0 (N/A)	4.2×10^{8} (47)
	indoor corncob burning	3.8×10^{10} (12)	0 (N/A)	3.8×10^{10} (14)
	indoor brush wood burning	6.2×10^{10} (9)	0 (N/A)	6.2×10^{10} (10)
transportation	gasoline	1.9×10^{10} (19)	3.97×10^{8}	5.58×10^{2}
	diesel	2.0×10^{11} (3)	5.36×10^{8}	2.23×10^{3}
	biogas	4.4×10^{9} (27)	1.26×10^{7}	4.46×10^{1}
	aviation	4.1×10^{9} (29)	3.24×10^{7}	6.52×10^{1}
	shipping	1.1×10^{8} (49)	1.23×10^{5}	9.14×10^{-1}
agriculture	gas/diesel	2.6×10^{10} (17)	4.9×10^{9} (17)	3.0×10^{10} (17)
	agriculture waste burning	2.0×10^{10} (18)	0 (N/A)	2.0×10^{10} (19)
	fertilizer application and livestock management	1.4×10^{11} (6)	0 (N/A)	1.4×10^{11} (7)

Supplementary Table 2 List of 42 economic sectors for the China multi-regional input-output model (MRIO) table

sector number	sector name
01	Agriculture, Forestry, Animal Husbandry and Fishery
02	Mining and washing of coal
03	Extraction of petroleum and natural gas
04	Mining and processing of metal ores
05	Mining and processing of nonmetal and other ores
06	Food and tobacco processing
07	Textile industry
08	Manufacture of leather, fur, feather and related products
09	Processing of timber and furniture
10	Manufacture of paper, printing and articles for culture, education and sport activity
11	Processing of petroleum, coking, processing of nuclear fuel
12	Manufacture of chemical products
13	Manufacture of non -metallic mineral products
14	Smelting and processing of metals
15	Manufacture of metal products
16	Manufacture of general purpose machinery
17	Manufacture of special purpose machinery
18	Manufacture of transport equipment
19	Manufacture of electrical machinery and equipment
20	Manufacture of communication equipment, computers and other electronic equipment
21	Manufacture of measuring instruments
22	Other manufacturing and waste resources
23	Repair of metal products, machinery and equipment
24	Production and distribution of electric power and heat power
25	Production and distribution of gas
26	Production and distribution of tap water
27	Construction
28	Wholesale and retail trades
29	Transport, storage, and postal services
30	Accommodation and catering
31	Information transfer, software and information technology services
32	Finance
33	Real estate
34	Leasing and commercial services
35	Scientific research
36	Polytechnic services
37	Administration of water, environment, and public facilities
38	Resident, repair and other services
39	Education
40	Health care and social work

41	Culture, sports, and entertainment
42	Public administration, social insurance, and social organizations

Supplementary Table 3 The regional background mortality rate (B) and integrated exposure-response (IER) model parameters (α, β, γ, and z_{cf}) for each of the five disease-burden causes, including ischemic heart disease (IHD), cerebrovascular disease (Stroke), chronic obstructive pulmonary disease (COPD), and lung cancer (LC) for adults over 25 years old, and acute lower respiratory infections (ALRI) for children under five. B is reported in deaths per 100000.

		IHD	Stroke	COPD	LC	ALRI
	East China	196	228	70.2	80.8	1.73
B	Middle China	214	250	80.1	71.8	2.28
	West China	157	232	143	61.9	7.59
α		2.54	1.31	22.5	86.9	2.42
β		8.07×10^{-2}	1.20×10^{-2}	1.12×10^{-3}	2.40×10^{-4}	1.46×10^{-2}
γ		4.66×10^{-1}	1.27	6.94×10^{-1}	7.78×10^{-1}	8.87×10^{-1}
z_{cf}		7.50	7.36	7.34	4.19	4.19

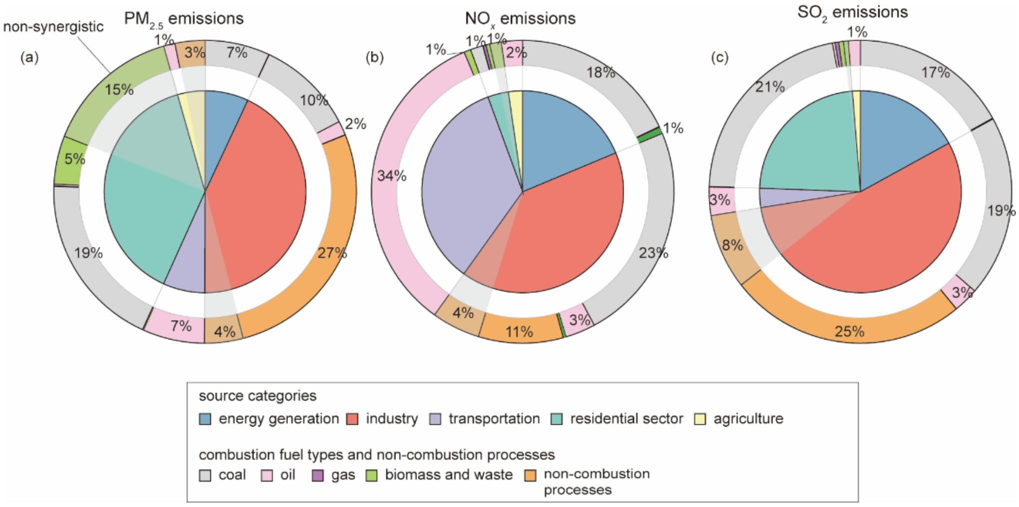

Supplementary Figure 1. Source attributions of (a) fine particulate matter ($PM_{2.5}$), (b) nitrogen oxides (NO_x), and (c) sulfur dioxide (SO_2) emissions. The inside pie charts represent the contribution of different sectors and the outside rings represent the contribution of different fuel types for combustion sources and non-combustion processes. The shaded areas are non-synergistic sources without direct carbon dioxide emissions.

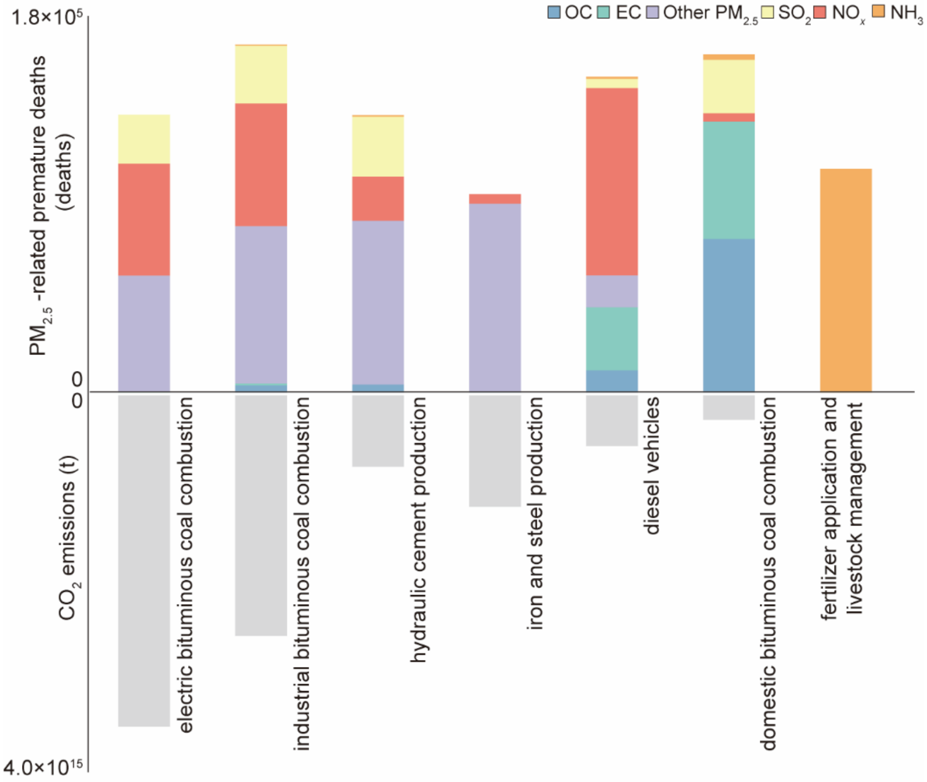

Supplementary Figure 2. Speciated contribution to fine particulate matter ($PM_{2.5}$) exposure-related premature deaths and carbon dioxide (CO_2) emissions from top contributors to premature deaths. The chart shows source contributions to $PM_{2.5}$ exposure- related premature deaths and CO_2 emissions from the seven subsectors with the highest contribution to premature deaths, including electric bituminous coal combustion, industrial bituminous coal combustion, hydraulic cement production, iron and steel production, diesel vehicles, domestic bituminous coal combustion, and fertilizer application and livestock management. The stacked bars show the speciated contributions from emissions of primary $PM_{2.5}$, including organic carbon (OC), elemental carbon (EC), and other primary $PM_{2.5}$, as well as gas precursors, including nitrogen oxides (NO_x), sulfur dioxide (SO_2), and ammonia (NH_3).

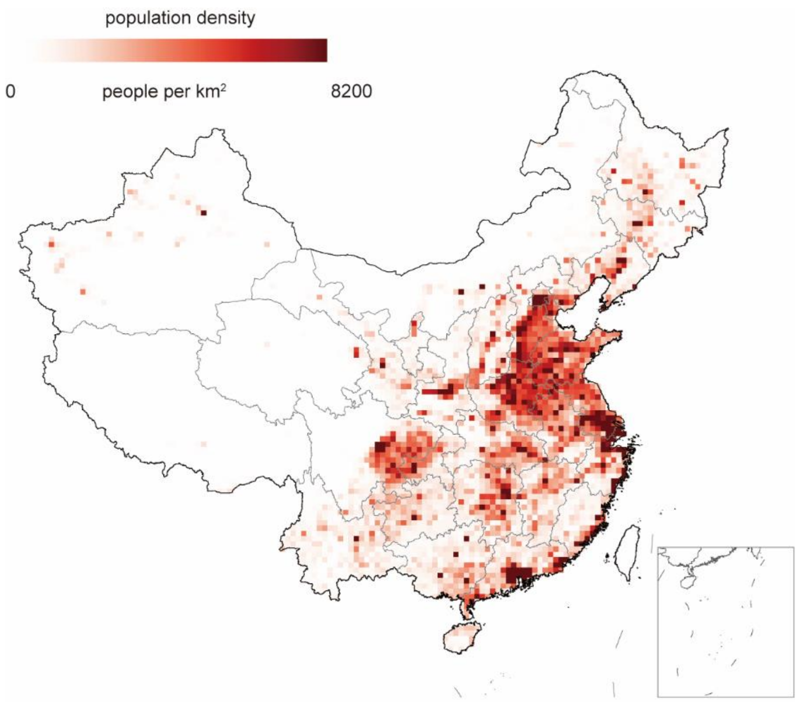

Supplementary Figure 3. Distribution of population density in China. The map displays population density at a 36 × 36 km resolution for grids in mainland China, as premature deaths attributable to long-term ambient $PM_{2.5}$ exposure in mainland China were defined as the cost function for adjoint simulations. The provincial boundary shapefile is obtained from Harvard Dataverse (https://doi.org/10.7910/DVN/DBJ3BX) and is publicly available under the Creative Commons CC0 Public Domain Dedication.

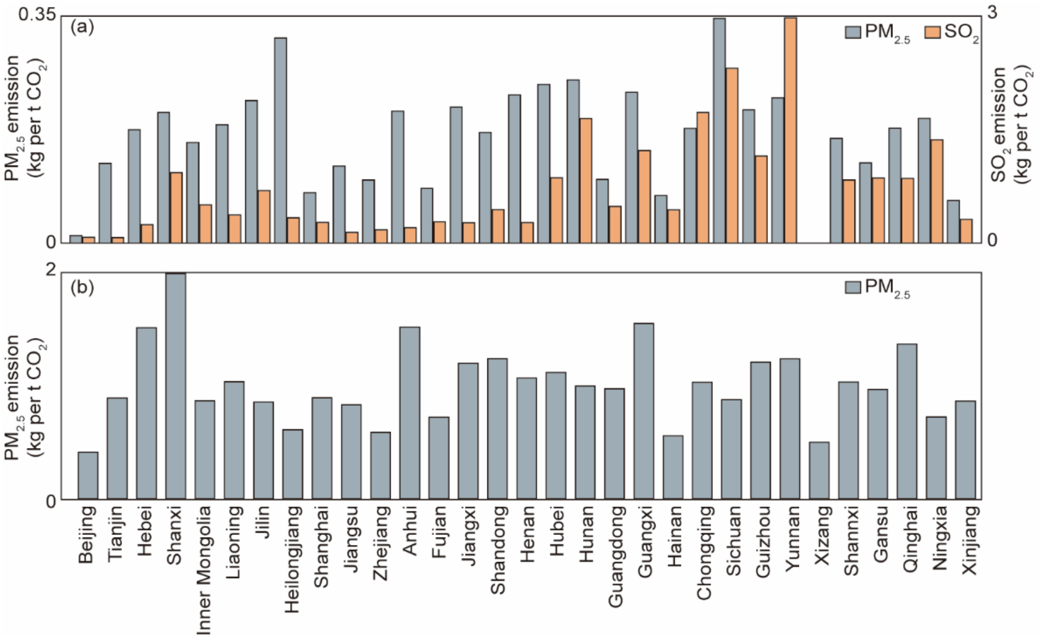

Supplementary Figure 4. Intensity of air pollutant emissions. (a) fine particulate matter ($PM_{2.5}$) and sulfur dioxide (SO_2) emission per ton carbon dioxide (CO_2) emission from bituminous coal combustion in the energy generation sector. (b) $PM_{2.5}$ emission per ton CO_2 emission from hydraulic cement production.

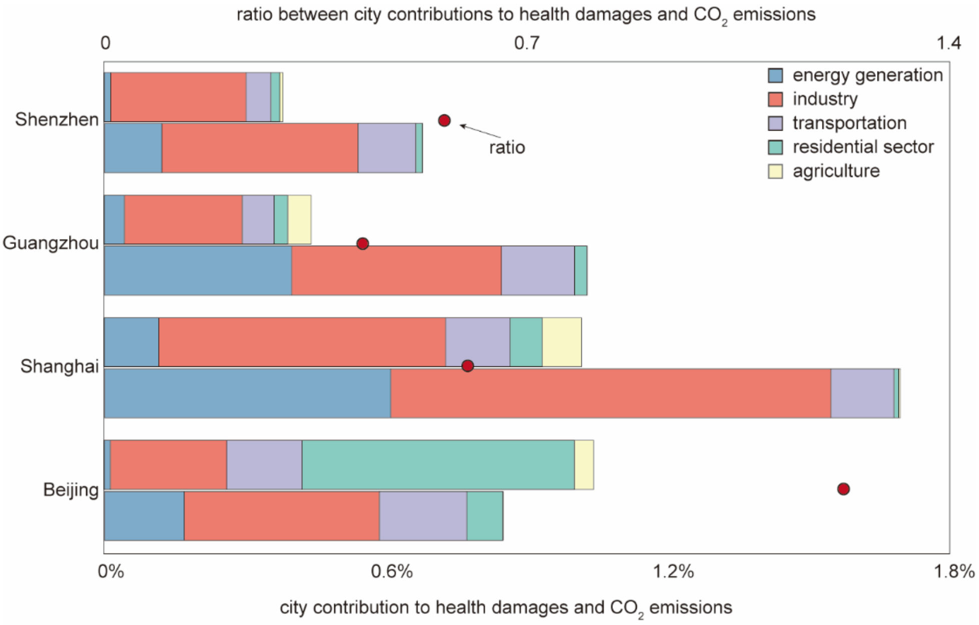

Supplementary Figure 5. Comparison of city contribution to fine particulate matter ($PM_{2.5}$)-related health damages and carbon dioxide (CO_2) emissions from top megacities in China. The stacked bars show source contributions to nationwide health damages (upper bars) and CO_2 emissions (lower bars) in each city. The red dots show the ratio between city contributions to health damages and CO_2 emissions.

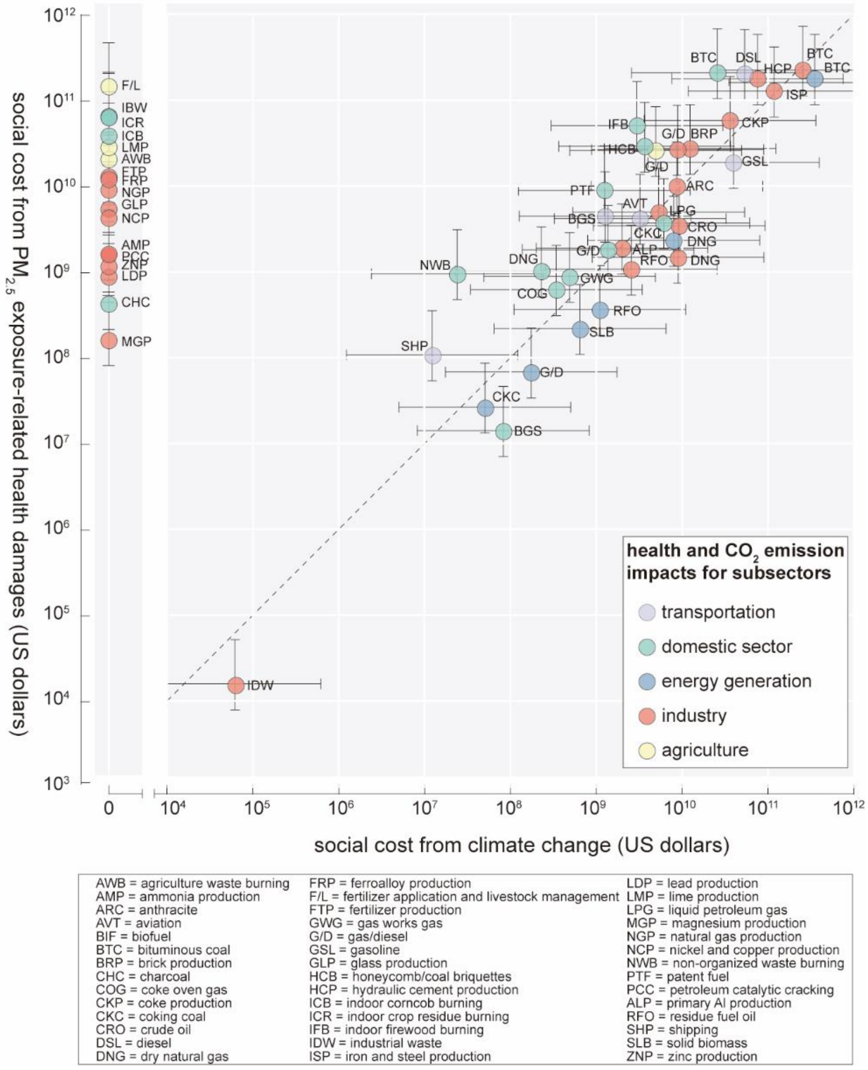

Supplementary Figure 6. The uncertainty range of sectoral monetized health damage and monetized climate impacts. The vertical error bars represent high and low end estimates of monetized health damage derived with high and low estimates of value of statistical life. The horizontal error bars represent high and low estimates of monetized climate impacts derived with high and low estimates of direct social cost of carbon.

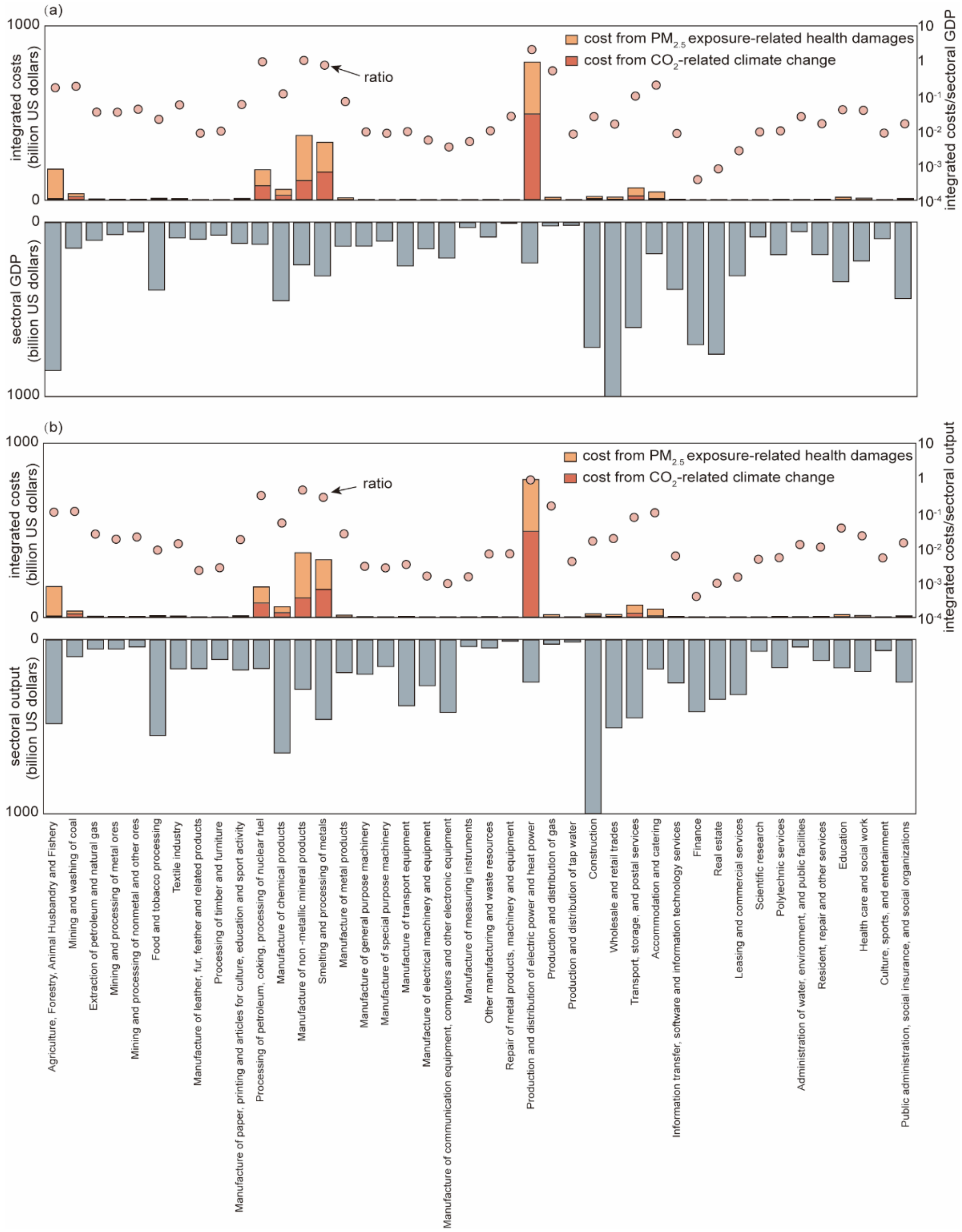

Supplementary Figure 7. Comparison between integrated costs and sectoral economic outputs. (a) comparison between integrated costs and sectoral gross domestic product (GDP). The stacked bars in the upper panel show integrated costs from $PM_{2.5}$ exposure-related health damages and CO_2-related climate change for the 42 economic sectors. The bars in the lower panel show the corresponding sectoral GDP in 2011 US dollars. The dots display ratio between integrated costs and sectoral GDP on log scale (minor axis). (b) comparison between integrated costs and sectoral output. The stacked bars in the upper panel show integrated costs and the bars in the lower panel show the corresponding sectoral output. The dots display ratio between integrated costs and sectoral output on log scale (minor axis).

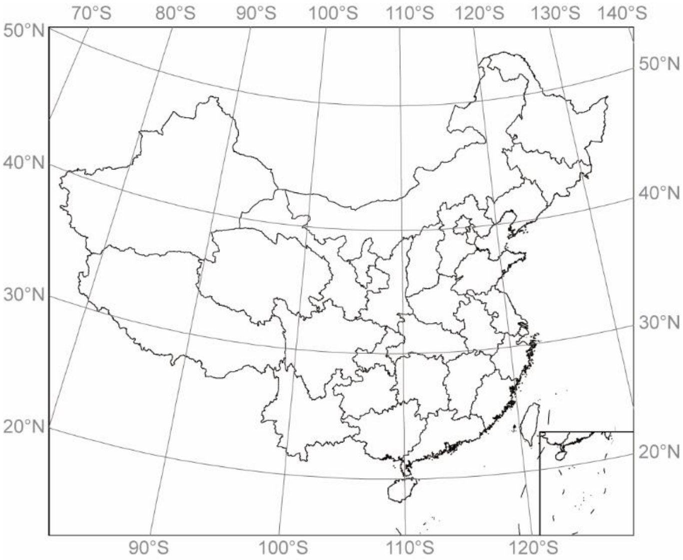

Supplementary Figure 8. The model domain for the Community Multiscale Air Quality (CMAQ) and CMAQ adjoint simulations in this study. The model domain and geographical location of each province in China are displayed. The provincial boundary shapefile is obtained from Harvard Dataverse (https://doi.org/10.7910/DVN/DBJ3BX) and is publicly available under the Creative Commons CC0 Public Domain Dedication.

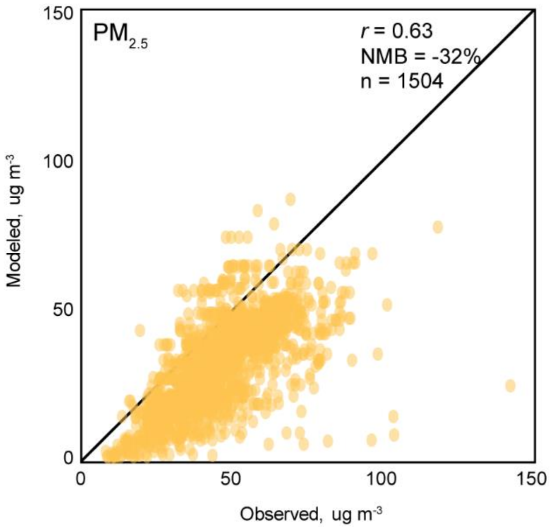

Supplementary Figure 9. Model evaluation for the Community Multiscale Air Quality (CMAQ) simulated $PM_{2.5}$ across China in 2017. Comparison between the CMAQ simulated and observed annual averaged $PM_{2.5}$ at 1504 sites in China. Pearson correlation coefficients (r), normalized mean bias (NMB), and sample size (n) are provided in the panel.

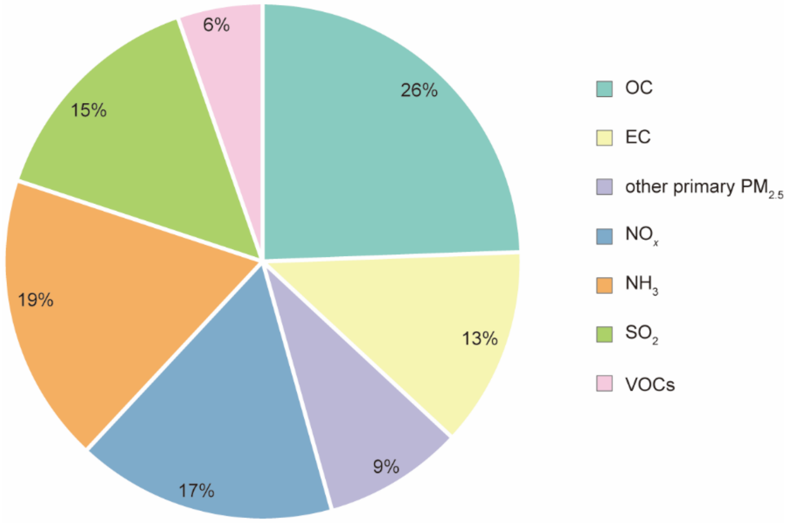

Supplementary Figure 10. Speciated contribution to $PM_{2.5}$-related mortality from emissions of primary fine particulate matter ($PM_{2.5}$) and precursors. The speciated contributions to $PM_{2.5}$-related mortality from primary $PM_{2.5}$ emissions and the emissions of precursors were calculated based on the adjoint sensitivity simulated by the CMAQ adjoint model and the gridded emissions. The semi-normalized sensitivities were calculated as the contributions of organic carbon (OC), elemental carbon (EC), and other primary $PM_{2.5}$, as well as gas precursors, including nitrogen oxides (NO_x), sulfur dioxide (SO_2), ammonia (NH_3), and volatile organic compounds (VOCs).

Supplementary Note 1

Due to the difference for sector classification between the PKU-FUEL inventory and the China multi-regional input-output model (MRIO) table [1], we assigned the production-based emissions to the 42 economic sectors (**Supplementary Table 2**) based on the source mapping process described below. Emissions from the electric sector, agriculture, and transportation sector were directly mapped to the corresponding sectors in the MRIO. Emissions from industrial combustion were attributed to the mining and manufacturing sectors utilizing provincial energy balance sheets [2] and sectoral energy consumption [3] as proxies. Emissions from industrial processes were mapped to the corresponding MRIO sectors based on the products categories. Emissions from the commercial sectors were split from the domestic subsectors based on the provincial energy balance sheets [2]. In the lack of sector-specific energy statistics, the commercial emissions were attributed to the tertiary industry sectors using sectoral monetary outputs in the MRIO table as proxies. Residential emissions cannot "flow" in trade, and were thus excluded in the emission mapping process.

Supplementary References

1. Zheng, H. et al., Chinese provincial multi-regional input-output database for 2012, 2015, and 2017. *Sci. Data* **8**, 244, doi:10.1038/s41597-021-01023-5 (2021).

2 National Bureau of Statistics of China (ed.), China energy statistical yearbook 2018. China Statics Press: Beijing (2018).

3 National Bureau of Statistics of China (ed.), China statistical yearbook 2018. China Statics Press: Beijing (2018).
