# Supplementary material for: Substantial differences in source contributions to carbon emissions and health damage necessitate balanced synergistic control plans in China
Source: Nat Commun. 2024 Jul 13;15:5880. doi: 10.1038/s41467-024-50327-8 (PMC11245606; doi:10.1038/s41467-024-50327-8)
Supplement: Supplementary file 3 — Reporting Summary [file 41467_2024_50327_MOESM3_ESM.pdf]

nature portfolio

Corresponding author(s): Yilin Chen

Last updated by author(s): Mar 6, 2024

Reporting Summary

Nature Portfolio wishes to improve the reproducibility of the work that we publish. This form provides structure for consistency and transparency in reporting. For further information on Nature Portfolio policies, see our Editorial Policies and the Editorial Policy Checklist.

Statistics

For all statistical analyses, confirm that the following items are present in the figure legend, table legend, main text, or Methods section.

n/a	Confirmed	
☐	☒	The exact sample size (n) for each experimental group/condition, given as a discrete number and unit of measurement
☒	☐	A statement on whether measurements were taken from distinct samples or whether the same sample was measured repeatedly
☒	☐	The statistical test(s) used AND whether they are one- or two-sided<br>*Only common tests should be described solely by name; describe more complex techniques in the Methods section.*
☒	☐	A description of all covariates tested
☒	☐	A description of any assumptions or corrections, such as tests of normality and adjustment for multiple comparisons
☒	☐	A full description of the statistical parameters including central tendency (e.g. means) or other basic estimates (e.g. regression coefficient) AND variation (e.g. standard deviation) or associated estimates of uncertainty (e.g. confidence intervals)
☒	☐	For null hypothesis testing, the test statistic (e.g. F, t, r) with confidence intervals, effect sizes, degrees of freedom and P value noted<br>*Give P values as exact values whenever suitable.*
☒	☐	For Bayesian analysis, information on the choice of priors and Markov chain Monte Carlo settings
☒	☐	For hierarchical and complex designs, identification of the appropriate level for tests and full reporting of outcomes
☐	☒	Estimates of effect sizes (e.g. Cohen's d, Pearson's r), indicating how they were calculated

Our web collection on statistics for biologists contains articles on many of the points above.

Software and code

Policy information about availability of computer code

Data collection	no software was used
Data analysis	The CMAQ Adjoint model (https://github.com/USEPA/CMAQ_ADJOINT) is utilized to simulate backward emission sensitivities. MATLAB R2021a was used for source attribution analysis in this study. The source codes utilized for source attribution in this study can be assessed on https://doi.org/10.5281/zenodo.11632297

For manuscripts utilizing custom algorithms or software that are central to the research but not yet described in published literature, software must be made available to editors and reviewers. We strongly encourage code deposition in a community repository (e.g. GitHub). See the Nature Portfolio guidelines for submitting code & software for further information.

Data

Policy information about availability of data

All manuscripts must include a data availability statement. This statement should provide the following information, where applicable:

- Accession codes, unique identifiers, or web links for publicly available datasets
- A description of any restrictions on data availability
- For clinical datasets or third party data, please ensure that the statement adheres to our policy

Demographic data used in this study can be accessed via https://landscan.ornl.gov/. Other data supporting health damage assessment and monetized social cost assessments are available within the article and Supplementary Information. China Multi-Regional Input–Output Table is available at http://www.ceads.net/data/

input_output_tables/. Datasets for gridded contribution to social costs from health damage and CO2-related climate change can be accessed on https://doi.org/10.5281/zenodo.10781209. Source data are provided with this paper.

Research involving human participants, their data, or biological material

Policy information about studies with human participants or human data. See also policy information about sex, gender (identity/presentation), and sexual orientation and race, ethnicity and racism.

Reporting on sex and gender	This study does not involve human participants or human data.
Reporting on race, ethnicity, or other socially relevant groupings	This study does not involve human participants or human data.
Population characteristics	This study does not involve human participants or human data.
Recruitment	This study does not involve human participants or human data.
Ethics oversight	This study does not involve human participants or human data.

Note that full information on the approval of the study protocol must also be provided in the manuscript.

Field-specific reporting

Please select the one below that is the best fit for your research. If you are not sure, read the appropriate sections before making your selection.

☐ Life sciences ☐ Behavioural & social sciences ☒ Ecological, evolutionary & environmental sciences

For a reference copy of the document with all sections, see nature.com/documents/nr-reporting-summary-flat.pdf

Ecological, evolutionary & environmental sciences study design

All studies must disclose on these points even when the disclosure is negative.

Study description	This study was based on a modeling framework that included the CMAQ-Adjoint model and source attribution analysis with coordinated emission inventories for PM2.5-related air pollutants and CO2. Treatment factors and interactions, design structure, nature and number of experimental units and replicates are irrelevant to this study.
Research sample	Existing datasets involved in this study are described in the "Data availability" section as follows: Demographic data used in this study can be accessed via https://landscan.ornl.gov/. Other data supporting health damage assessment and monetized social cost assessments are available within the article and Supplementary Information. China Multi-Regional Input–Output Table is available at http://www.ceads.net/data/input_output_tables/. Emission inventories are available at https://gems.sustech.edu.cn/home. This study does not involve sample or organism taxa.
Sampling strategy	Our study involved air quality modeling based on publicly available datasets, with no sampling procedure.
Data collection	No new data were collected; we relied entirely on these existing, publicly available datasets.
Timing and spatial scale	No new data were collected; we relied entirely on these existing, publicly available datasets.
Data exclusions	No data were excluded from our analysis.
Reproducibility	The findings of our study are reproducible, provided that the same model setup, emission inventory, and meteorological inputs are used.
Randomization	This study did not involve randomization, as it was not relevant to the study design. This study's source attribution approach directly links emissions to health impacts with atmospheric dispersion and chemical transport model. Thus, the nature of this study does not involve covariate control.
Blinding	Our study analyzed publicly available data using emission inventories, air quality modeling, and IER functions, with no experimental groups or interventions. The nature of the study did not include group allocation or subjective assessments that could be influenced by investigator bias. Therefore, blinding was not relevant.

Did the study involve field work? ☐ Yes ☒ No

Reporting for specific materials, systems and methods

We require information from authors about some types of materials, experimental systems and methods used in many studies. Here, indicate whether each material, system or method listed is relevant to your study. If you are not sure if a list item applies to your research, read the appropriate section before selecting a response.

Materials & experimental systems

n/a	Involved in the study
☒	☐ Antibodies
☒	☐ Eukaryotic cell lines
☒	☐ Palaeontology and archaeology
☒	☐ Animals and other organisms
☒	☐ Clinical data
☒	☐ Dual use research of concern
☒	☐ Plants

Methods

n/a	Involved in the study
☒	☐ ChIP-seq
☒	☐ Flow cytometry
☒	☐ MRI-based neuroimaging

Plants

Seed stocks	This is not relevant to our study.
Novel plant genotypes	This is not relevant to our study.
Authentication	This is not relevant to our study.
